# Protein Expression Profiling Identifies Key Proteins and Pathways Involved in Growth Inhibitory Effects Exerted by Guggulsterone in Human Colorectal Cancer Cells

**DOI:** 10.3390/cancers11101478

**Published:** 2019-10-01

**Authors:** Rari Leo, Lubna Therachiyil, Sivaraman K. Siveen, Shahab Uddin, Michal Kulinski, Joerg Buddenkotte, Martin Steinhoff, Roopesh Krishnankutty

**Affiliations:** 1 Translational Research Institute, Academic Health System, Hamad Medical Corporation, Doha 3050, Qatar; RLeo@hamad.qa (R.L.); LTherachiyil@hamad.qa (L.T.); SSivaraman@hamad.qa (S.K.S.); SKhan34@hamad.qa (S.U.); MKulinski@hamad.qa (M.K.); JBuddenkotte@hamad.qa (J.B.); MSteinhoff@hamad.qa (M.S.); 2 Department of Pharmaceutical Sciences, College of Pharmacy, Qatar University, Doha 2713, Qatar; 3 Department of Dermatology and Venereology, Hamad Medical Corporation, Doha 3050, Qatar; 4 Department of Medicine, Weill Cornell Medicine-Qatar, Qatar Foundation-Education City, Doha 24144, Qatar; 5 Department of Medicine, Weill Cornell Medicine, 1300 York Avenue, New York, NY 10065, USA; 6 College of Medicine, Qatar University, Doha 2713, Qatar

**Keywords:** colorectal cancer, HCT 116, SW620, guggulsterone, label-free shotgun proteomics, intrinsic apoptosis pathway, NF-kB signaling

## Abstract

Colorectal cancer (CRC) is a leading killer cancer worldwide and one of the most common malignancies with increasing incidences of mortality. Guggulsterone (GS) is a plant sterol used for treatment of various ailments such as obesity, hyperlipidemia, diabetes, and arthritis. In the current study, anti-cancer effects of GS in human colorectal cancer cell line HCT 116 was tested, potential targets identified using mass spectrometry-based label-free shotgun proteomics approach and key pathways validated by proteome profiler antibody arrays. Comprehensive proteomic profiling identified 14 proteins as significantly dysregulated. Proteins involved in cell proliferation/migration, tumorigenesis, cell growth, metabolism, and DNA replication were downregulated while the protein with functional role in exocytosis/tumor suppression was found to be upregulated. Our study evidenced that GS treatment altered expression of Bcl-2 mediated the mitochondrial release of cytochrome c which triggered the formation of apoptosome as well as activation of caspase-3/7 leading to death of HCT 116 cells via intrinsic apoptosis pathway. GS treatment also induced expression of p53 protein while p21 expression was unaltered with no cell cycle arrest. In addition, GS was found to inhibit NF-kB signaling in colon cancer cells by quelling the expression of its regulated gene products Bcl-2, cIAP-1, and survivin.

## 1. Introduction

Colorectal cancer (CRC) is one of the most prevalent malignancies worldwide with a significant cause of human mortality [1,2]. CRC is ranked third most common cancer in the world with approximately one million new cases being diagnosed annually [3,4]. An early detection of the disease convinces a 5-year survival rate of about 90%, while late diagnosis at advanced stages drastically brings down the survival time nearly to 11 months [5]. The conventional treatment of CRC by chemotherapy still remains challenging as non-specificity of chemotherapeutics by targeting not only tumor specific cells but also, non-malignant cells can result in many side effects, eventually leading to multi-drug resistance. This kind of cytotoxic therapy can impair the quality of patient’s life and undesirably affect the course, outcome as well as costs of the treatment. Complementary and alternative medicines that serve as better therapeutics which could significantly improve the management of colorectal cancer are hence in demand. Natural compounds (phytochemicals), the biologically active substances derived from plants, hold great potential for medicinal applications even in cancer therapeutics.

Guggulsterone (GS) [4, 17(20)-pregnadiene-3, 16-dione] is a plant sterol extracted from the gum resin of tree *Commiphora mukul* used for treatment of various ailments such as obesity, hyperlipidemia, diabetes, and arthritis [6]. GS has been reported to inhibit proliferation, suppress invasion, angiogenesis, tumor initiation, promotion, and metastasis in cancer cells [7]. Notably, resistance to growth inhibition exerted by GS in normal human fibroblasts, non-transformed prostate and colon epithelial cell lines in comparison to cancer cells makes it an interesting drug to explore in the context of finding alternative anticancer agents for better cancer therapeutics [8,9]. Though various mechanisms have been proposed in explaining the anticancer effects of GS, mainly by binding to the farnesoid X receptor [10] and modulating the expression of antiapoptotic proteins, its mechanism of action in colorectal cancer cells still remains elusive. Colorectal cancer forms a model system to study human tumors as epithelial cells of colon mucosa follows a systematic cellular process of proliferation, differentiation, and adenoma formation, eventually transforming into a malignant tumor [11]. In addition, studies have demonstrated that correlating the mRNA and protein expression to predict specific protein expression levels using quantitative mRNA data can be biased which indicates the drawback of using transcript level expression alone for analysis and hence, conducting expression analysis at protein level could be more informative [12,13].

Proteomics can be defined as the large-scale comprehensive study of a specific proteome which forms the set of all proteins expressed in a cell or a biological system or organism at a given time and condition [14]. In the context of colorectal cancer research, proteomic studies have been carried out specifically to find proteins that could serve as biomarkers for disease diagnosis and also to identify proteins involved in molecular pathways leading to cancer metastasis and progression [15]. Advances in mass spectrometry-based proteomics has enabled the technique by using a variety of labeling and label-free approaches to quantify the differential abundance of proteins in cells, tissues, tumors, and even body fluids. One of the most widely used mass spectrometry based proteomics approach is label-free shotgun proteomics which is effective for in-depth protein identification as well as in obtaining the global proteome profiles [16].

In the current study, we primarily investigated the growth inhibitory effects of GS in human colorectal cancer cell lines HCT 116 (luminal) and SW620 (metastatic). We performed a comparative proteome profiling of GS treated vs. untreated cells using label-free proteomic profiling based on shotgun proteomics approach. Our study divulged some of the novel proteomic signatures from GS treated HCT 116 cells with their differential expression indicating that GS significantly reduced the cell proliferation/migration, cell growth and metabolism, carcinogenesis, as well as DNA replication whereas enhanced the process of exocytosis/tumor suppression. Our data suggests that GS treatment altered expression of Bcl-2 mediated the mitochondrial release of cytochrome c which triggered the formation of apoptosome as well as activation of caspase-3/7 leading to cell death of HCT 116 cells via intrinsic apoptosis pathway. Our study results provide a comprehensive view on the mechanism of action of GS in colorectal cancer cells which could mark its anticancer potential and its beneficial use as a therapeutic agent in future for clinical applications.

## 2. Results

### 2.1. Diffeential Inhibition of Cell Proliferation by GS in HCT 116 and SW620 Cell Lines

**Figure 1:**
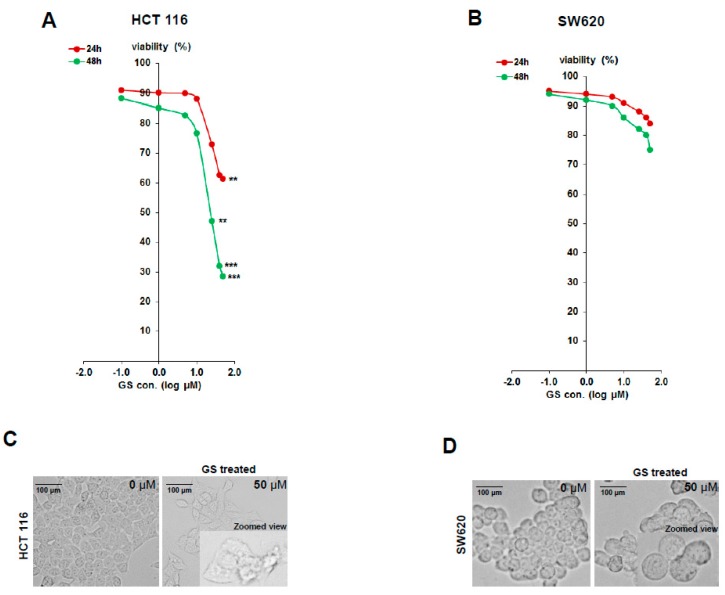
Guggulsterone inhibits proliferation of HCT 116 cells. Cell viability was determined in HCT 116 and SW620 cells after 24 and 48 h of incubation with varying concentration of Guggulsterone (GS). Viable cell counts were determined by MTT (3-(4,5-dimethylthiazol-2-yl)-2,5-diphenyltetrazolium bromide) assay. Plots show dose- and time-response of (**A**) HCT 116 and (**B**) SW620 cells % viability with GS treatment. Data are presented as mean ± SD (*n* = 6). Significance was calculated using Student’s *t*-test; ** *p* < 0.005, *** *p* < 0.003. (**C**,**D**) Effect of GS treatment on morphology of colorectal cancer cell lines. HCT 116 cells (C) and SW620 (D) were treated with GS (50 µM) for 48 h. Images were captured under a light microscope under 40× magnification. Zoomed in image is attached in frame of GS treated picture plate. GS treatment resulted in significant morphological changes in HCT 116 cells with loss of cell integrity as well as blebbing compared to the untreated intact cells.

To evaluate the effect of GS on cell viability of colon cancer cells, HCT 116 (derived from colon adenocarcinoma) and SW620 (derived from colon adenocarcinoma metastasis to lymph node) were treated with increasing doses of GS for 24 h and 48 h and cell viability was determined by MTT (3-(4,5-dimethylthiazol-2-yl)-2,5-diphenyltetrazolium bromide) reduction assay. GS induced dose as well as time-dependent inhibition of cell proliferation in both the cell lines while, viable cells population was significantly reduced (28%) in HCT 116 cells compared to SW620 (61%) at 48 h (Figure 1A,B). The IC_50_ value of GS for HCT 116 was determined to be 21 µM. The effect of GS treatment on HCT 116 cells were more promising than SW620 with the drug exhibiting significant cytotoxicity to HCT 116 cells as evident from (Figure 1A,B). Morphologically the HCT 116 cells were more confluent with intact cell membrane before treatment with GS while after the treatment, significant decrease in confluency was observed and the cells were found to lose their integrity along with blebbing as well as forming cytoplasmic vesicles (Figure 1C). In case of SW620 cells, though GS treatment resulted in a limited decrease in confluency, no significant changes in the cell membrane integrity was observed (Figure 1D) and this could be due to the limited sensitivity of the cells to GS.

Since GS exhibited significant growth inhibition on HCT 116 cells, we intended to explore the differential expression that can occur at protein level as a result of the drug treatment. For this, we performed a comparative proteomic profiling of the GS treated HCT 116 cells vs. untreated using mass spectrometry-based label-free proteomics approach as illustrated in Supplementary Figure S2.

### 2.2. Proteomic Profiling of GS Treated HCT 116 Cells

To identify the differentially expressed proteins in GS treated HCT 116 cells we used a label-free quantitative shotgun proteomics method. To cope with the biological and experimental variations we included triplicate runs in LC-MS/MS analysis with samples from three independent replicates. The Pearson correlation analysis of the LFQ intensities of proteins identified from untreated (*r* = 0.874) and treated (*r* = 0.885) replicate samples (Supplementary Figure S1A,B) showed a strong positive correlation accounting to a reproducible, relative label-free quantification between replicates.

**Figure 2:**
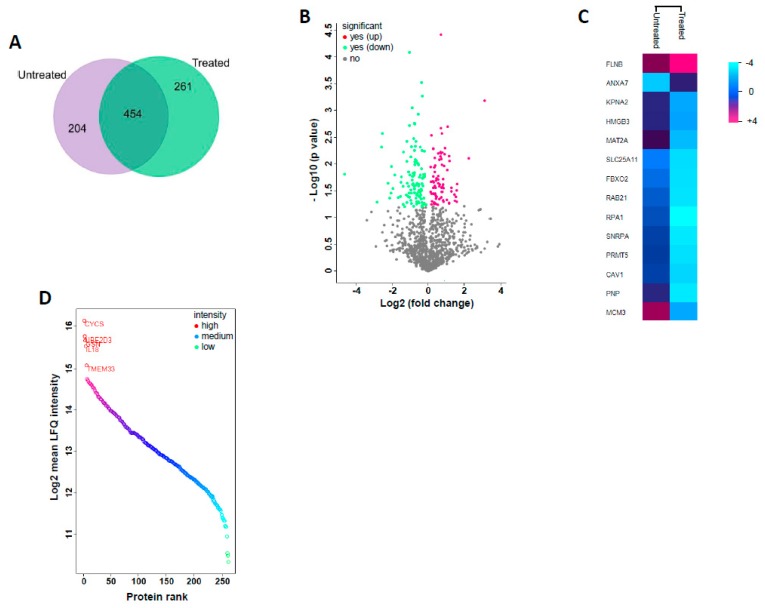
Proteomics data interpretation and visualization. (**A**) Venn diagram showing the unique proteins identified in untreated vs. guggulsterone treated samples and the overlapping proteins are the ones present in both. (**B**) Volcano plot comparing differential protein expression in untreated and GS treated samples. The proteins significantly upregulated are red dots and downregulated are green dots, while the grey dots represent the proteins with unaltered expression. The *p*-value < 0.05 was used for this significance cutoff. (**C**) Heatmap of the significantly dysregulated proteins after GS treatment with FDR (*p*-value) < 0.05 as significance threshold and fold-change >2 or <−1.5 as differential abundance threshold. (**D**) Dynamic range of quantified proteins uniquely present in GS treated sample. Distribution of expression intensities of quantified proteins show a large dynamic range of abundance with five proteins (CYCS, UBE2D3, GSN, IL8, and TMEM33) exclusively expressed with very high orders of magnitude.

Proteomics analysis allowed an overall identification of 919 proteins of which 658 were in untreated and 715 in GS treated cells with 454 proteins in common (Figure 2A). To identify the differentially expressed proteins from GS treatment, a label-free quantification approach using the MaxQuant label-free algorithm (LFQ) that measures the relative protein abundance based on spectral intensity was employed. To determine significant differences between the GS treated and untreated samples, the LFQ intensities of each protein groups were compared using *t*-test. With FDR <0.05 as significance threshold and fold-change >2 or <−1.5 as differential abundance threshold, we found 14 proteins to be significantly dysregulated in the GS treated samples compared to untreated (Table 1). The comparative analysis of proteomes from guggulsterone treated and untreated cells is graphically represented as a volcano plot (Figure 2B) drawn using the fold-change and the *p*-value obtained from *t*-tests displaying significant differences between the groups. An unsupervised hierarchical clustering of the differential expressed proteins based on LFQ intensities is represented as a heatmap (Figure 2C). The unique proteins in guggulsterone treated sample sorted based on their decrementing LFQ intensities resulted in identifying four proteins with high abundance as represented in Figure 2D. The data from comparative proteomics analysis can be found in Supplementary Table S1.

### 2.3. Functional Annotation of Dysregulated Proteins

**Figure 3:**
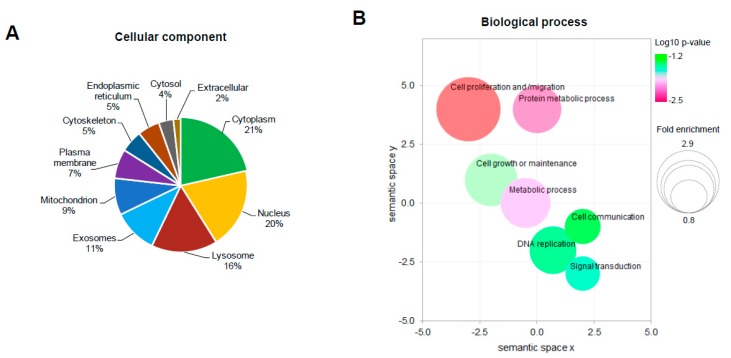
Functional annotation and classification of dysregulated proteins by GS treatment. The top enriched terms from the functional enrichment analysis of the dysregulated proteins and their distribution in the categories of cellular component (**A**) as a pie diagram, biological process (**B**) as bubble plot. Statistics of functional enrichment analysis can be found in Supplementary Table S2.

The functional annotation of dysregulated proteins (DEPs) was performed using FunRich (functional enrichment analysis) tool. The topmost enriched terms in each categories: cellular component and biological processes are as represented in Figure 3. Broadly, the most enriched terms in cellular component category were cytoplasm (21%) and nucleus (20%) (Figure 3A), while the biological process category showed ‘cell proliferation and/migration’, ‘protein metabolic process’, as well as ‘cell growth or maintenance’ as the most enriched terms (Figure 3B). The dysregulated proteins were found to be mainly involved in DNA binding, cytoskeletal protein binding, as well as phosphorylase, transferase, and transporter activities, as these were the most enriched terms under the molecular function category (Supplementary Table S2).

### 2.4. Proteomic Signatures of GS Treated HCT 116 Cells

**Figure 4:**
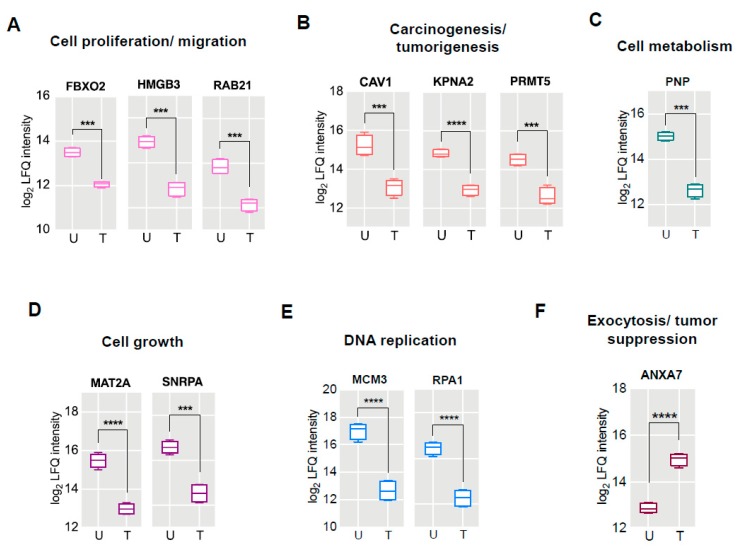
Proteomic signatures of GS treated HCT 116 cells. Box plots representing the differential expression of proteins based on abundance (LFQ intensity) in untreated and treated samples categorized based on their functional roles (**A**) cell proliferation/migration, (**B**) carcinogenesis/tumorigenesis, (**C**) cell metabolism, (**D**) cell growth, (**E**) DNA replication, and (**F**) exocytosis/tumor suppression. U: untreated, T: GS treated. *** *p* < 0.002; **** *p* < 0.0001.

The comparative proteomic profiling of guggulsterone treated vs. untreated cells followed by label-free quantification resulted in identifying 14 proteins which were significantly dysregulated (Table 1). The dysregulated proteins were classified into categories based on their functional role and their differential abundance calculated from the differences in log_2_ LFQ intensity. The proteins F-box only protein 2 (FBXO2), high mobility group protein B3 (HMGB3), and Ras-related protein Rab-21 (RAB21) involved in cell proliferation/migration were found to be downregulated by almost 2-fold (Figure 4A). The proteins with potential role in inducing carcinogenesis/tumorigenesis were also identified, which included caveolin-1 (CAV1), importin subunit alpha-1 (KPNA2) and protein arginine N-methyltransferase 5 (PRMT5) were >2-fold downregulated (Figure 4B). The other proteins identified to be less abundant (>2-fold expression) included purine nucleoside phosphorylase (PNP) involved in cell metabolism (Figure 4C) while S-adenosylmethione synthase isoform type-2 (MAT2A) and U1 small nuclear ribonucleoprotein A (SNRPA) were known to mediate cell growth (Figure 4D). The proteins with functional roles in DNA replication process were identified to be dysregulated, which included: DNA replication licensing factor MCM3 (MCM3) and replication protein A 70 kDa DNA-binding subunit (RPA1) were downregulated by 4.6-fold and 2.3-fold respectively (Figure 4E). The protein Annexin A7 (ANXA7) having functional role in tumor suppression was found in high abundance, almost 2-fold upregulated (Figure 4F) in GS treated cells.

### 2.5. GS Treatment Reduced Cell Proliferation and Migration in CRC Cells

**Figure 5:**
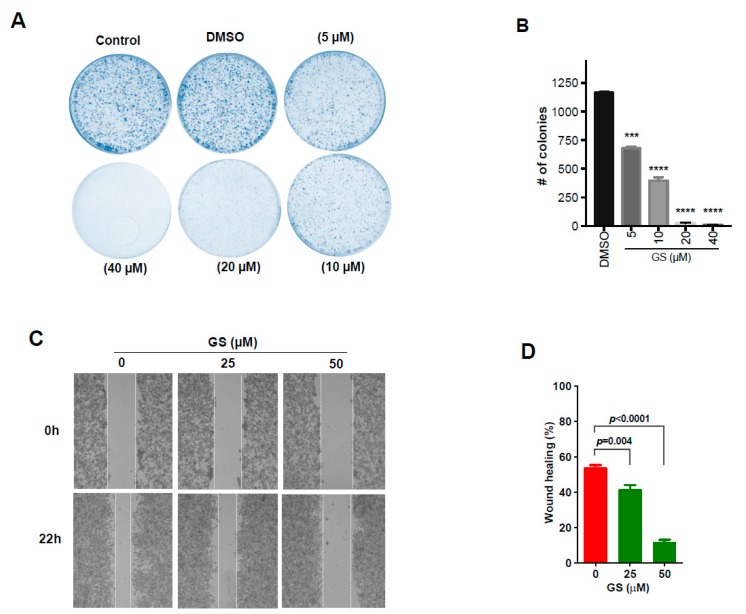
GS significantly reduced the proliferation and migration of HCT 116 cells. (**A**) Colony formation assay of HCT 116 cells, wherein cells were treated with different doses of GS (as indicated) or vehicle (DMSO) for 2 weeks and stained with crystal violet. Images were captured by a CCD camera and are representative of three independent experiments. (**B**) Colony formation of HCT 116 cells. Dose-dependent effect of GS in colony formation of HCT 116 cells; data represented as mean ± SD from three replicate experiments. (**C**) Wound healing assay; HCT 116 cells grown to confluency, wounded (t = 0 h) by a sterile pipette tip and then treated with different concentrations (25 and 50 µM) of GS. Cells were observed under a light microscope after 22 h of incubation and imaged. (**D**) Percentage of wound healing relative to the distance measured in images in (C) quantified using image J. Values are represented as mean ± SD. Data are representative of triplicate experiments. *** *p* < 0.001; **** *p* < 0.0002.

We used clonogenic and wound healing assays to validate the functional role (with the proteomic signatures being grouped as mentioned above) assessed to be modulated by GS treatment. The clonogenic assay showed a dose-dependent effect in the formation of colonies with a significant decrease in number of colonies being observed with incrementing dose of GS (Figure 5A,B). A significant reduction in wound healing was observed in GS treated HCT 116 cells, with a wound closure of 15% at the highest concentration tested compared to the untreated (Figure 5C,D), implying reduction in cell migration. Taken together, these results validate the anti-proliferative and anti-migratory effects of GS in CRC cells.

### 2.6. Pathways Enriched in GS Treated HCT 116 Cells and Their Validation by Protein Arrays

**Figure 6:**
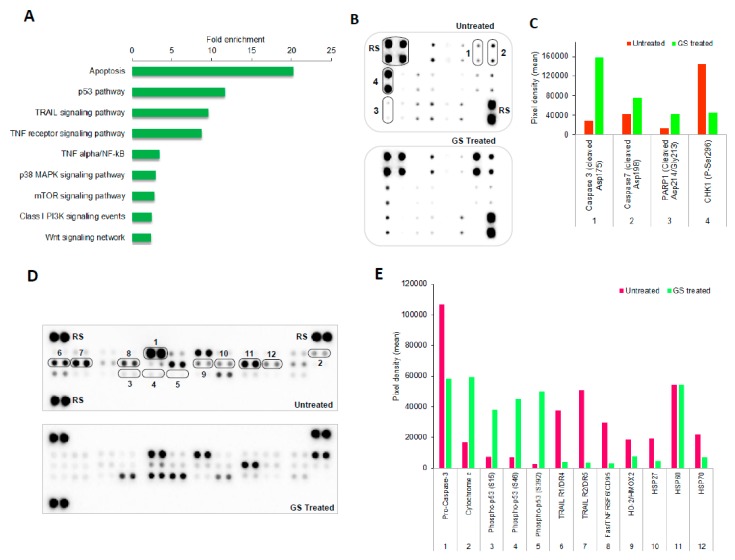

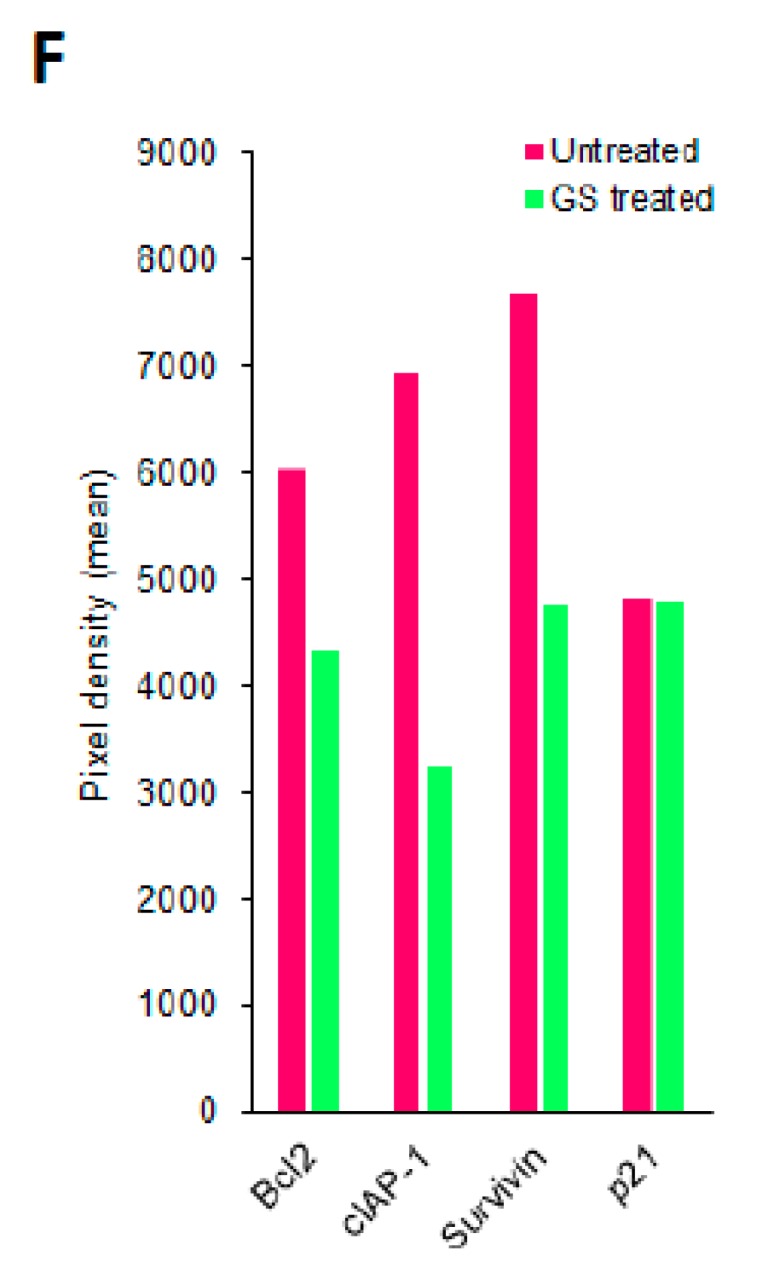
Biological pathways enriched by GS treatment and their validation by antibody arrays. (**A**) The top nine pathways enriched by the functional enrichment analysis of the dysregulated proteins. (**B**,**D**) Representative images of the antibody arrays showing expression levels of various proteins: (**B**) Human apoptosis signaling array C1 (RayBiotech) and (**D**) Proteome profiler Human apoptosis array (R&D Systems). (**C**,**E**,**F**) Quantitative profiles of protein expression levels on antibody arrays by densitometry analysis. Values are represented as mean ± SD (*n* = 6). Images are representative of three independent experiments. RS denotes reference spots. The arrays are spotted with the antibodies in duplicates. The array coordinate maps with the corresponding antibody/protein names are as illustrated in Supplementary Figures S3 and S4.

The functional enrichment analysis of dysregulated proteins using FunRich tool resulted in identifying various signaling pathways in GS treated HCT 116 cells. The topmost enriched pathways are represented in Figure 6A which included mainly apoptosis, p53, TRIAL (TNF-related apoptosis-inducing ligand), and TNF (tumor necrosis factor) receptor signaling pathways as well as TNF-alpha/NF-kB (nuclear factor kappa-light-chain-enhancer of activated B cells). To validate these pathways, the cell extracts from HCT 116 cells treated with or without GS were analyzed by protein arrays such as Human Apoptosis Signaling Array C1 (Ray Biotech) and Proteome Profiler Human Apoptosis array kit (R&D systems).

The changes in expression levels of various apoptotic related proteins in treated and untreated cells from both the protein array analysis are as shown in Figure 6B,D. Cleavage of PARP (poly ADP ribose polymerase), caspase-3 and caspase-7 proteins were observed from analysis of the human Apoptosis Signaling Array C1 (Ray Biotech) with the GS treated sample compared to the untreated one (Figure 6B,C). To validate more of the enriched signaling pathways, the Proteome Profiler Human Apoptosis Array Kit (R&D systems) was used. With this array an increased expression of cytochrome c along with lowered expression of pro-caspase-3 was observed with the GS treated sample (Figure 6D,E). Taken together the cleavage of PARP, the cleaved caspases (3/7) along with the differential expression of cytochrome c and pro-caspase-3 validates the apoptosis signaling. The levels of p53 proteins of various phosphorylation (S15, S46, and S392) were found to be elevated in GS treated cells while, the protein levels of TRAIL receptors as well as TNF receptor were lowered (Figure 6D,E) validating the involvement of p53, TRAIL, and TNF receptor pathways. The heat shock proteins heme oxygenase-2 (HO-2), Hsp27, and Hsp70 essential for cell survival were found to be decreased in expression in GS treated cells (Figure 6D,E) compared to the untreated. The HSP60 (housekeeping) protein level served as the internal control. The anti-apoptotic proteins Bcl-2, cIAP-1, and survivin (NF-kB target genes) were found to be suppressed in their expression with the GS treatment as evidenced from the antibody array analysis (Figure 6F) implying the role of NF-kB which was one among the most enriched pathways.

### 2.7. GS Treatment Induced Apoptosis in HCT 116 Cells

**Figure 7:**
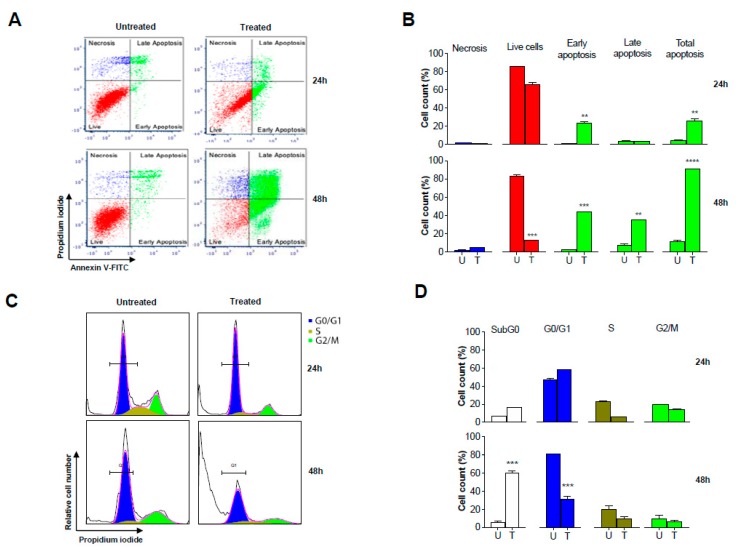
GS induced apoptosis in HCT 116 cells but no cell cycle arrest. HCT 116 cells were left untreated or treated with GS (50 µM) for 24 and 48 h. (**A**) The cells were then stained with Annexin V FITC and propidium iodide (PI) and analyzed by flow cytometry for apoptosis. (**B**) Percent distribution of necrosis, live cells, and apoptosis at 24 h and 48 h. (**C**) The cells were stained with PI and DNA content analyzed by flow cytometry to determine the cell cycle distribution. (**D**) Percent distribution of the cells on phases of cell cycle. ** *p* < 0.005, *** *p* < 0.001, **** *p* < 0.0002.

To further validate the apoptosis induced by GS treatment as evidenced from the antibody array analysis, the HCT 116 cells treated with GS for 24 h and 48 h were analyzed by flow cytometry. Figure 7A, B shows a time-dependent decrease in the live cell population of HCT 116 cells with GS treatment while, apoptosis was evident as the percent of cells progressively increased from early apoptosis through late apoptosis accounting to a total apoptosis of more than 80% after 48 h. On the other hand, with the cell cycle analysis, the majority of the cells were found to accumulate in sub-G0/G1 phase after 48 h of GS treatment with a time-dependent decrease in the G0/G1 population (Figure 7C). The increase in the subG0/G1 phase population could be accounted for the apoptotic fraction of cells further confirming apoptosis with no cell cycle arrest (Figure 7D).

### 2.8. Validation of Proteomic Signatures and Key Proteins From Antibody Array by Western Blot Anlaysis

**Figure 8:**
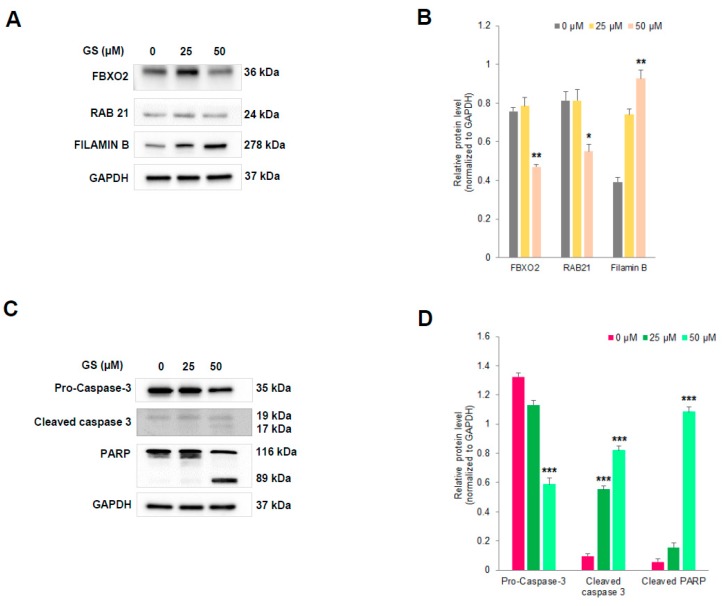
Validation by western blot analysis. (**A**,**C**) HCT 116 cells were treated with increasing doses of GS (as indicated) for 48 h and cells were lysed for western blot analysis with the antibodies FBXO2, RAB21, Filamin B, pro-caspase-3, cleaved caspase 3, and PARP. GAPDH served as the loading control. (**B**,**D**) Relative protein levels quantified by densitometry analysis. * *p* < 0.03, ** *p* < 0.002, *** *p* < 0.0001.

To further verify the differential expression of proteins induced by GS treatment in HCT 116 cells identified by proteomic analysis, we tried to validate randomly picked proteins from Table 1 by western blot analysis. We found almost a 2-fold decrease in level of expression of FBXO2 and RAB21 proteins and more than a 2-fold increase in expression of Filamin B protein (Figure 8A,B) with increasing dose of GS treatment which were well in agreement with the differential expression observed by proteomics analysis.

In addition, some of the key proteins involved in apoptosis found to be differentially expressed after GS treatment in HCT 116 cells by antibody array analysis were also validated by western blot analysis. As evident from Figure 8C,D, GS treatment resulted in a dose-dependent decrease in expression of Pro-caspase3 along with cleavage of caspase 3 as well as PARP indicating the induction of apoptosis. These data were, again, well in agreement with what was observed by antibody array analysis (Figure 7C,E).

## 3. Discussion

Guggulsterone (GS) has been proven to impose anticancer effects on many types of cancer cells including colon cancer through many of proposed mechanisms [17]. Though these mechanisms explaining the anti-cancerous effects imposed by GS were proposed, the underlying processes and molecules involved are yet unknown and needs to be elucidated. The present study describes the mechanism through which GS induces anticancer effects in colorectal cancer HCT 116 cells identified by a label-free comparative proteomics profiling technique. Our results from the initial growth inhibitory studies showed that GS exerted a stronger growth inhibitory effect on human colorectal cancer HCT 116 cells (luminal) compared to the SW620 (metastatic) cells as evidenced by the percent decrease in cell viability estimated by the proliferation assay. Significant inhibition of cell growth by GS in HCT 116 cells was observed with cell viability reduced to 28% after 48 h of treatment (at 50 µM concentration). It is noteworthy that the metastatic cell line SW620 exhibited limited sensitivity to GS treatment resulting in less growth inhibition compared to HCT 116 cells and pursuing the underlying mechanism of this limited sensitivity is certainly of future interest.

Since significant growth inhibition was observed in HCT 116 cells by GS treatment, we sought to explore the underlying mechanism. Proteomics technology makes it possible to have a comprehensive characterization of various molecular orchestrations that can occur in cells by profiling the protein expression patterns thereby identifying the key molecules involved in cellular processes. We used state-of-the-art proteomics technology, liquid chromatography coupled to tandem mass spectrometry (LC-MS/MS) based shotgun proteomics approach to identify and characterize the modulated molecular events in HCT 116 cells with GS treatment. We performed a comparative proteomic profiling of the GS treated vs. untreated HCT 116 cells and evaluated differential expression of proteins based on abundance measured by label-free quantification technique (Figure 2).

The proteomic profiling resulted in a total of 919 proteins being identified of which 658 were in untreated and 715 in GS treated cells while, 454 proteins were found to be commonly present. A label-free quantification approach that measures the relative protein abundance based on spectral intensity was used to identify the dysregulated proteins. With an FDR <0.05 as significance threshold and a fold-change >2 or <−1.5 as differential abundance threshold, fourteen proteins were found to be significantly dysregulated in HCT 116 cells with guggulsterone treatment (Table 1). Eighty percent of these dysregulated proteins were found to be downregulated after GS treatment. Functional annotation of the dysregulated proteins using functional enrichment analysis resulted in identifying their localization by cellular component classification and the biological processes, as well as molecular functions they are involved in. The majority of proteins (about 21%) identified were cytoplasmic as the whole cell lysate was used for proteomics analysis. The rest of the proteins were found to be localized in nucleus, lysosomes, mitochondrion, cytoskeleton, and endoplasmic reticulum (Figure 3A). Cell proliferation and migration, protein metabolic process, cell growth or maintenance, as well as DNA replication were the most enriched biological processes. This implies that these processes known to be active in cancer cells were modulated by GS treatment in HCT 116 cells. DNA binding, cytoskeletal protein binding along with phosphorylase, transferase and transporter activities were the most enriched molecular functions which again are hallmarks of the cancer-specific proteins.

Comparative proteomic profiling using label-free quantitative approach resulted in identifying significantly dysregulated proteins that could be regarded as the proteomic signatures of GS treated HCT 116 cells. We grouped these proteomic signatures according to their functional roles in colorectal cancer, thereby classifying them into six different functional categories (Figure 4). Increased cell proliferation and migration are typical properties of cancer cells which leads to tumor formation as well as metastasis. From our study we identified three proteins: FBXO2, HMGB3, and RAB21 with 2-fold lowered expression after GS treatment, which were functionally involved in cell migration/metastasis. F-box only protein 2 (FBXO2), also known as FBG1 or Fbs1, is a member of the FBXO protein family that broadly falls into the category of F-box proteins that recognizes high-mannose-type asparagine-linked carbohydrate chains (N-glycans) [18]. The study by Xinying Wei et al., [19] has reported the prognostic significance of FBXO2 in colorectal cancer, as FBXO2 was highly expressed in colorectal cancer and the authors suggests its use as a biomarker for cell proliferation and metastasis. High mobility group box 3 (HMGB3) is a member of high-mobility group box (HMGB) family and the study by Zheying Zhang et al., [20], showed that HMGB3 promotes growth and migration in colorectal cancer. The same study has showed the involvement of HMGB3 in carcinogenesis and development of colorectal cancer mediated via WNT/β-catenin pathway and suggests HMGB3 to be a promising therapeutic target of colorectal cancer. Ras-related protein Rab-21 (RAB21) belongs to the family of Rab proteins which are small GTPases involved in the traffic of endocytotic vesicles [21]. Although no reports exists on the role of RAB21 in colorectal cancer, the study by Teijo Pellinen et al. [21], has shown that over-expression of RAB21 stimulates cell migration and cancer cell adhesion to collagen and human bone. Significant downregulation observed with these three proteins—FBXO2, HMGB3, and RAB21—suggests that GS treatment significantly reduced cell proliferation and migration of HCT 116 cells. Significant reduction in colony formation (Figure 5B) and cell migration (Figure 5D) of GS treated HCT 116 cells as evident from the functional assays, such as clonogenic and wound healing, further confirms the anti-proliferative and anti-migratory effects exerted by GS.

Carcinogenesis/tumorigenesis forms hallmark of colorectal cancer progression which leads to formation of colon adenocarcinomas. Our study identified three different proteins CAV1, KPNA2, and PRMT5 known to be involved in inducing tumorigenesis and they were downregulated by more than 2-fold after GS treatment. Caveolin-1 (CAV1) is one of the principal proteins of Caveolin family that forms the major structural proteins of caveolae, the vesicular invaginations of the plasma membrane [22]. Cav-1 has been reported to play an important role in the progression of carcinoma [23] as well as involved in multiple cancer-associated processes such as tumor growth, cell migration/metastasis, cell death and survival, multidrug resistance (MDR), and angiogenesis [24]. Fine et al., [25] has studied the expression of caveolin-1 immunohistochemically in paraffin-embedded sections of normal epithelium, adenoma, and adenocarcinoma, and found its expression to be limited or nearly absent in normal colonic epithelium but significantly elevated in colonic adenocarcinomas. Another study by Patlolla et al., [26], examined the expression of caveolins at protein and mRNA levels in rat colon adenocarcinoma vs. adjacent normal mucosa and found caveolin-1 being over-expressed in colon adenocarcinoma. The authors have also verified caveolin-1 overexpression at protein and mRNA level in human colon cancer cell lines HT-29 and HCT 116. Karyopherin alpha 2 (KPNA2) belongs to the family of karyopherin, the nuclear transport proteins; any aberrant expression or dysfunction of these transporter proteins were shown to be associated with tumorigenesis and tumor progression [27,28]. Studies have revealed that elevated expression of KPNA2 can be associated to colorectal cancer progression [29], can be used as a novel diagnostic and prognostic marker as well as a therapeutic target for colorectal cancer [30,31]. Protein arginine methyltransferase 5 (PRMT5) is a member of PRMTs that catalyze the symmetric demethylation of arginine residues of histone H3 and H4 which in turn alters chromatin structure leading to transcriptional repression [32]. The studies by Baolai Zhang et al., [33] and Lakshmi Prabhu et al., [34] evidenced that PRMT5 was overexpressed in colorectal cancer cells which promoted cancer progression, while its expression in patient-derived primary tumors correlated with increased cell growth and decreased overall patient survival. We identified three proteins of tumorigenesis: CAV1, KPNA2, and PRMT5 being downregulated by GS treatment in colorectal cancer HCT 116 cells.

The protein purine nucleoside phosphorylase (PNP) catalyzes the reversible phosphorolysis of purine nucleosides (inosine and guanosine) in humans which forms a part of purine metabolism [35]. We found a 2-fold decrease in expression of this protein in GS treated HCT 116 cells. PNP is considered as a therapeutic target in malignant lymphoproliferative diseases as treatment with its inhibitor Forodesine (BCX-1777) induced apoptosis of chronic lymphocytic leukemia (CLL) cells [36,37]. The study by Kojima et al., has shown that the miRNAs miR-I and miR-I33a targets PNP gene which potentially functions as an oncogene favoring oncogenesis and progression of prostate cancer (PCa) and hence, suggests PNP inhibition as a novel strategy for better treatment of PCa [38]. In our study, we found PNP expression to be significantly downregulated by GS treatment in HCT 116 cells indicating that it forms one of the potential targets of GS in this colorectal cancer cells.

The other proteins that were found to be significantly downregulated after the GS treatment in HCT 116 cells included MAT2A (methionine adenosyltransferase 2A), SNRPA (U1 small nuclear ribonuceloprotein A), MCM3 (DNA replication licensing factor MCM3), and RPA1 (replication protein A 70 kDa DNA-binding subunit). Among them, MAT2A and SNRPA were found to be involved in cell growth, with studies showing increased MAT2A expression in human colon cancer being implicated in the pathogenesis of colon cancer [39]. Recently, SNRPA has been identified as potential oncogene in gastric cancer (GC), with its expression in tumor tissues being a factor for proliferation efficiency [40]. The study showed that high SNRPA expression enhances tumor cell growth in GC as well as poor prognosis in GC patients. The other proteins MCM3 and RPA1 with more than 2-fold lowered expression after GS treatment were found to be involved in DNA replication. Recent studies have shown that elevated expression of MCM3 in tumor tissues of hepatocellular carcinoma patients predicted worse overall survival [41], while RPA1 being demonstrated as a candidate oncogene which influences tumor biological behaviors in many cancers including colon cancer [42].

Annexin A7 (also known as synexin) is a member of annexin family which is involved in exocytosis including hormone secretion in glandular tissues [43,44]. The functional role of annexin A7 in different cancers is still controversial as some of the data indicate that it functions as a tumor-suppressor gene in prostate cancer, melanoma, however might act as tumor promoter in gastric cancer, liver and breast cancer [45]. A study by Duncan et al., [46], wherein authors tried to profile the expression of annexins in colorectal cancer, could not detect annexin A7 expression either in normal colon or colorectal cancer. At the same time, annexin A7 has been proposed as a putative tumor suppressor gene in prostate cancer [47] while, a recent study revealed that annexin A7 expression was downregulated in late-stage gastric cancer and is negatively correlated with the differentiation grade and apoptosis [48]. From our current study, we found a significant upregulation of annexin A7 protein expression in HCT 116 cells treated with GS, indicating that GS treatment in this colorectal cancer cells leads to increased expression of annexin A7 and is positively correlated with apoptosis.

The functional enrichment analysis of dysregulated proteins resulted in identifying various signaling pathways in GS treated HCT 116 cells. The topmost enriched pathways included: apoptosis, p53, TRIAL, and TNF receptor signaling pathways as well as TNF-alpha/NF-kB (Figure 6A). To further validate the enriched pathways we used Proteome Profiler antibody arrays: Human Apoptosis Signaling Array C1 (RayBiotech) and Proteome Profiler Human Apoptosis Array Kit (R&D systems) to determine the involvement of related proteins. The critical proteins implicated with cell death and apoptotic pathways were found to be dysregulated in GS treated HCT 116 cells as observed from protein array analysis.

Apoptosis is a programmed process of cellular self-destruction [49] triggered by two different pathways namely extrinsic [50] and intrinsic [51]. The intrinsic pathway (so-called Bcl-2 regulated) is activated by stress conditions followed by mitochondrial dysfunction events leading to apoptosis while, the apoptosis in extrinsic pathway (so-called death receptor) is activated by ligation of members of the tumor necrosis factor receptor (TNRF) family bearing intracellular death domain. In both the pathways, activation of caspases and degradation of antiapoptotic proteins are involved in inducing cell death.

From our data of proteome profiler array analysis, the key protein that play a critical role in intrinsic apoptosis, cytochrome c was found to be significantly expressed in GS treated HCT 116 cells, while the expression of antiapoptotic proteins Bcl-2, cIAP-1, and survivin were downregulated. The pro-caspase-3 levels were observed to be decreasing along with cleavage of caspase-3, caspase-7, and PARP (Figure 6C,E). Also with GS treatment, we found the heat shock proteins such as heme oxygenase-2 (HO-2), Hsp27, and Hsp70 essential for cell survival being downregulated in HCT 116 cells. Taken together, these data indicate that GS treatment in HCT 116 cells altered expression of Bcl-2 mediated the mitochondrial release of cytochrome c, which triggered the formation of apoptosome as well as activation of caspase-3/7 leading to cell death via intrinsic apoptosis pathway. Studies had indicated that activation of death receptors—such as Fas, TRAILs, TNFR, etc.—mediate extrinsic apoptosis pathway [52,53]. In our study, we found that GS inhibited the expression of these death receptors which confirms no involvement of extrinsic pathway in the induction of apoptosis in HCT 116 cells.

Studies had revealed p53 (tumor suppressor protein) mediated induction of apoptosis interlinked with the mitochondrial pathway via regulation of Bcl-2 or via cell cycle arrest through p21 protein overexpression [54]. The data from our study showed enhanced expression of three major phosphorylated p53 proteins: p53 S15, S46, and S392 (Figure 6E) while the expression of p21 protein was found to be unaltered (Figure 6F). Hence, data from our study confirms p53-mediated induction of intrinsic apoptosis in GS treated HCT 116 cells. The flow cytometry analysis demonstrating the induction of apoptosis in GS treated HCT 116 cells with total apoptosis accounting to 80% with no cell cycle arrest being observed is in support to the above findings. In addition, NF-kB signaling, one of the major pathways known to be constitutively activated in many cancers was found to be inhibited by GS in HCT 116 cells from the current study, as lowered expression of Bcl-2, cIAP-1, and survivin (the NF-kB target genes) were observed with GS treatment.

Our data indicates that GS treatment in HCT 116 cells leads to induction of p53-mediated intrinsic apoptosis resulting in significant cell death. GS was found to significantly reduce the cell proliferation and migration as well as inhibit the NF-kB signaling. The LC-MS/MS based label-free proteomics approach facilitated comprehensive profiling of protein expression changes, thereby confidently identifying the dysregulated proteins that can be regarded as the proteomics signatures of GS treated colon cancer HCT 116 cells. The data obtained from functional assays further enhanced the reliability of the GS targets we identified. The most enriched pathways identified by the functional enrichment analysis using proteomic signatures were further validated by proteome profiler antibody arrays. The differential protein expression profiles of the target proteins from the array further enhance the reliability on the mechanism of action of GS in colon cancer cells elucidated from the present study.

Though lack of known toxicity makes GS an interesting naturally-derived compound, its anticancer potential should be further more explored. Although the doses used in the current study can be achieved in vivo, its pharmacological potentiality will have to be evaluated by pharmokinetics as well as pharmodynamics studies. Hence, further studies in this aspect, as well as including in vivo animal models are of urgent value to ascertain the therapeutic value of this anticancer agent.

## 4. Materials and Methods 

### 4.1. Chemicals

The drug Z-Guggulsterone with purity >98% was purchased from Tocris Bioscience (Bristol, UK) and dissolved in dimethyl sulfoxide (DMSO) at a stock solution of 20 mM and stored at −20 °C. The stock solution was diluted in cell culture medium to the desired concentrations as indicated in each experiment.

### 4.2. Cell Lines and Culture Conditions

The human colon cancer cell lines HCT 116 and SW620 were obtained from the ATCC (Manassas, Virginia VA, USA). Cell lines were cultured in Dulbecco’s modified Eagle’s medium (DMEM) supplemented with 10% (v/v) fetal bovine serum (FBS) and antibiotics (penicillin 100 U/mL, streptomycin 10 μg/mL) at 37 °C in a humidified atmosphere of 5% CO_2_ and 95% air.

### 4.3. Cell Viability

The effect of GS on viability of colon cancer cells were measured by assay using the tetrazolium dye MTT. The assay was performed by seeding HCT 116 or SW620 cells (5 × 10^3^ cells /well) into a 96-well tissue culture plate and allowing them to attach overnight. The cells were then treated with indicated concentrations of drug or vehicle (DMSO) alone for the indicated time periods. Medium was then removed and 100 μL of 0.5 mg/mL MTT reagent (Thiazolyl Blue Tetrazolium Bromide; Sigma-Aldrich, St. Louis, Missouri MO USA) was added to the wells and incubated at 37 °C for 2 h. The MTT regent was then aspirated and 100 μL of isopropanol was added to dissolve the formazan crystal formed by viable cells. The plate was then incubated on a plate shaker for 1 h at room temperature before reading the A570 nm on a plate reader. Cell viability of each of the indicated treatments was expressed as a percent of the vehicle control.

### 4.4. Morphological Changes

To study morphological changes, the cell lines (5 × 10^5^ cells /well) were seeded into 6-well plate and incubated overnight to adhere. The cells were then treated with different concentrations of GS (25 and 50 μM). At 48 h post treatment the images were captured under the microscope (Nikon ECLIPSE, Tokyo, Japan) at 20× magnification.

### 4.5. Experimental Design

For the proteomics experiments, the cell culture was performed in 6-well plates and cells from three subsequent wells were pooled to obtain the required amount of protein per condition/sample. The proteomics study was performed in triplicates, in three independent experiments and in total of six biological replicates per condition were used for data analysis. HCT 116 cells (0.4 × 10^5^ cells/well) were seeded in the 6-well plate with complete DMEM media and incubated overnight at 37 °C with 5% CO_2_. The medium was changed after 24 h with fresh 2 mL (each well) of conditioned medium for control cells, conditioned media containing 0.05% DMSO (vehicle) for cells as control of treatment and conditioned medium containing 25 µM of GS for cells as treatment. Forty-eight hours post treatment, the cells were washed with cold PBS and lysed by adding cold 1× radioimmunoprecipitation assay (RIPA) buffer (20 mM Tris-HCL, pH 7.5, 150 mM NaCl, 1 mM Na_2_EDTA, 1 mM EGTA, 1% NP-40, 1% sodium deoxycholate, 2.5 mM sodium pyrophosphate, 1 mM β-glycerophosphate, 1 mM Na_2_VO_4_, 1 µg/mL leupeptin) supplemented with protease-phosphatase cocktail inhibitors (Roche). The cell lysate was obtained by collecting the supernatant after centrifugation at 14,000× *g* for 15 min at 4 °C and the protein content was quantified using Rapid Gold BCA Protein assay kit (Pierce^TM^, Thermo Scientific, Waltham, MA, USA).

### 4.6. Sample Preparation for Proteomics

Proteins (50 µg) from both untreated and GS treated cell lysates were separated using 4–15% Mini-PROTEAN^®^ TGX™ Precast Protein Gel (BioRad, Hercules, CA, USA), stained with Coomassie Brilliant Blue R-250 staining solution (BioRad, Hercules, CA, USA) for 1 h. The gel was then transferred into ultrapure water and stored at 4 °C overnight. Total proteins resolved using mono-dimensional gel electrophoresis was subjected to protein-in-gel digestion. Briefly, protein bands (12 gel slices) were excised and cut into 1 mm^3^ pieces destained, dehydrated with 90% acetonitrile (ACN), and rehydrated in 25 mM ammonium bicarbonate buffer (ABC). The proteins were reduced by adding 10 mM dithiothreitol (DTT) and alkylated by 55 mM iodoacetamide (IAA). The proteins were digested in ABC buffer containing sequencing grade modified trypsin (12.5 ng/µL) (Promega, Amdison, WI, USA) with trypsin to protein ratio of 1:100 for overnight at 37 °C. Peptides were extracted from the gel slices using 5% formic acid, 50% ACN. The solution was concentrated to near dryness under vacuum, redissolved in 5% formic acid and the peptides were prepared for mass spectrometry analysis using StageTips according to Rappsilber et al. [55].

### 4.7. Liquid Chromatography-Tandem Mass Spectrometry (LC-MS/MS) Analysis 

The peptides of each sample on Stage tip was desalted, concentrated by vacuum centrifugation and reconstituted in 10 µL of 5% formic acid. MS experiments were performed on a quadrupole-time of flight mass spectrometer (MS-QTOF 6550, Agilent Technologies, Santa Clara, CA, USA) coupled to a nano-liquid chromatography system (Agilent Technologies). The desalted peptides were separated on a 15 cm long C18 column with an inner diameter of 75 µm (Zorbax 300SB-C18, Agilent Technologies, Santa Clara, CA, USA) using gradient of buffer A (Ultrapure water, 1% formic acid) and buffer B (Acetonitrile, 1% formic acid) at a flowrate of 300 nL min-1. The chromatographic gradient was set to provide a linear increase from 2% to 80% buffer B in 110 min, for a total run time of 120 min. MS data was acquired on data dependent mode dynamically choosing the top ten most abundant precursor ions from the survey scan (400–1800 *m/z*) for fragmentation and MS/MS analysis. Precursors with a charged state of +1 were rejected and the dynamic exclusion duration was set as 25 s.

### 4.8. MS Data Processing and Analysis

The MS raw data were processed using MaxQuant software version 1.5.5.1 according to the standard workflow [56] with the built-in search engine Andromeda [57]. Proteins were identified by searching against the Uniprot human reference proteome (July 2019) database. Carbamidomethylation of cysteines was set as fixed modification, while protein N-terminal acetylation and methionine oxidation were defined as variable modifications for peptide search. The false discovery rates (FDR) for peptide and protein identifications were set to 1%. A maximum of two missed cleavages were allowed for tryptic digestion. The MaxLFQ label-free quantitation method [58] with retention time alignment and match-between-runs feature in MaxQuant was applied to extract the maximum possible quantification information. Protein abundance was calculated based on normalized spectral intensity (LFQ intensity).

Data analysis was performed using the freely available software Perseus (version 1.6.2.3) (https://maxquant.net/perseus/) [59]. The LFQ intensities from the MaxQuant analysis were imported, transformed by log2(×) and the missing LFQ intensity values were replaced (imputated) with the value of the lowest LFQ intensity from the normal distribution (width = 0.3, down shift = 1.8). The protein quantification and the statistical significance was calculated using two-tailed Student’s *t*-test with permutation-based FDR of 5% used for truncation of all test results. *p*-value < 0.05 with fold change ratio >2 or <−1.5 were considered to indicate significant protein abundance changes. The gene ontology (GO) annotation of the significantly dysregulated proteins carried out using FunRich (version 3.1.3) (http://www.funrich.org/) functional enrichment analysis tool [60] resulted in identifying the distribution of proteins involved with their enrichment in various categories such as cellular component, biological process, molecular functions, and biological pathways.

### 4.9. Clonogenic Assay

The effect of GS on colon cancer cells survival was evaluated by clonogenic assay according to the protocol of Franken et al. [61]. Briefly cells (1 × 10^4^ cells/well) were seeded in 6-well plate having complete DMEM media containing different concentrations of GS and incubated in CO_2_ incubator at 37 °C for 2 weeks. Medium with drug or vehicle was replaced twice a week. After treatment, the cells were washed with PBS, fixed with methanol, and stained with crystal violet solution. The excess stain was washed with ultrapure water, images were captured by CCD camera and colonies were counted using ImageJ software (https://imagej.nih.gov/ij/) [62,63]. Surviving fraction was calculated using the formula according to Franken et al. [61].

### 4.10. Wound Healing Assay

Cells were seeded at a density of 5 × 10^5^ cells/well into 6-well plate until they became 80% confluent. A scratch/wound was then made with a sterile 200 µL tip in each well of plate, washed and replaced with GS (25 and 50 μM) or vehicle containing medium. The plate was observed under microscope (Nikon ECLIPSE Ti-S, Tokyo, Japan) at 10 × magnification at 0 h and then incubated at 37 °C. After 22 h the plate was again observed under microscope. Images were captured at 0 and 22 h, analyzed using image J software for the distance of wound healing.

### 4.11. Protein Array Analysis

Cell lysate prepared from GS treated or untreated vehicle cells at 48 h post treatment was analyzed for apoptosis-related proteins using Human Apoptosis Signaling Array C1 (RayBiotech, Georgia GA, USA) and Proteome Profiler Human Apoptosis array kit (R&D systems, Minneapolis, MN, USA) according to the manufactures instructions. In brief, cell lysates from untreated and treated cells were prepared using lysis buffer from the respective array kits and protein concentration was measured by Rapid Gold BCA Protein assay kit (Pierce^TM^, Thermo Scientific, Waltham, MA, USA). Cell lysates diluted in array buffer were incubated with the ready-to-use pre-coated array membranes (blocked in blocking buffer provided with the kit) overnight at 4 °C on a rocking platform shaker. The array membranes were washed three times (10 min, 1 × interval) with washing buffer (provided with the kit) to remove any unbound proteins. The membranes were then incubated with the detection antibody cocktail for 1.5 h at room temperature while shaking. The membranes were again washed 3 × (5 min each) with wash buffer and further incubated with diluted streptavidin-HRP for 30 min at room temperature on shaking. The excess buffer was removed and the protein spots were detected by chemiluminescence by addition of Chemi Reagent mix (from the kit) while exposing for 1 min. The arrays were visualized, and images captured by a ChemiDoc^TM^ MP imaging system (BioRad, Hercules, CA, USA). The densitometric analysis of the protein array was performed using image J software with the Protein Array Analyzer plugin [64]. The pixel density of each duplicated protein spots in array was averaged and normalized against the reference spots and the relative levels were expressed as mean pixel intensity. The identity and coordinates of all the antibodies orientated on both the arrays are as described in Supplementary Materials.

### 4.12. Apoptosis Assay by Annexin V/PI Staining

To measure the apoptosis incidence, a commercial Annexin-V-FITC/PI kit (BD Biosciences, San Jose, CA, USA) was used according to manufacturer’s instructions. Briefly, HCT 116 cells were treated with or without the drug GS for 24 h and 48 h. Post treatment, the cells were collected by trypsinization, washed with cold PBS, and resuspended in binding buffer. The cells were then stained with Annexin V-FITC/PI and analyzed by flow cytometry using a BD LSRFortessa analyzer (BD Biosciences). Viable (live) cells are Annexin-V^neg^, PI^neg^; early apoptosis cells are Annexin-V^pos^, PI^neg^; late apoptosis cells are Annexin-V^pos^, PI^pos^ and Annexin-V^neg^, PI^pos^ are necrotic cells. The data was quantified and expressed as percent of the cell counts.

### 4.13. Cell Cycle Analysis

Approximately 8 × 10^5^ cells/well were seeded in a 6-well plate followed by overnight incubation at 37 °C (with 5% CO_2_). Cells were then treated with or without GS for 24 h and 48 h. Post treatment cells were harvested by centrifugation, fixed in 70% ice cold ethanol and kept at 4 °C overnight. Cells were then resuspended in PBS containing 25 µg/mL PI and 100 µg/mL RNAase, final concentrations and incubated at 37 °C for 30 min. The cell cycle profiles were analyzed by flow cytometry using a BD LSRFortessa analyzer (BD Biosciences). Data were analyzed with ModFit software (Verity Software House, Topsham, ME, USA) and represented as percent of cells distributed across the phases of cell cycle.

### 4.14. Western Blot Analysis

HCT 116 cells were treated with GS (25 and 50 µM) for 48 h and lysed with radioimmunoprecipitation assay (RIPA) lysis buffer. The lysates were centrifuged at 14,000× *g* for 10 min at 4 °C and supernatant collected. Protein concentration was measured using Rapid Gold BCA Protein assay kit (Pierce ^TM^, Thermo Scientific, Waltham, MA, USA). Thirty-five micrograms of total protein was separated by sodium-dodecyl sulfate polyacrylamide gel electrophoresis and transferred to polyvinylidene difluoride (PVDF) membrane and immunoblotted using antibodies: FBXO2, RAB21, Filamin B purchased from Invitrogen; Pro-Caspase-3, Cleaved Caspase 3, PARP, and GAPDH purchased from Cell Signaling Technology. The immunoreactive bands were detected using enhanced chemiluminescence solution (BioRad, Hercules, CA, USA) and visualized by a ChemiDoc^TM^ MP imaging system (BioRad, Hercules, CA, USA). The densitometric analysis of the protein array was performed using image J software.

### 4.15. Statistical Analysis

In proteomic analysis, the statistical significance of protein quantification was calculated using two-tailed Student’s *t*-test with permutation-based FDR of 5% used for truncation of all test results. Pearson correlation tests with continuity correction were employed to compare qualitative variables. For all other experiments statistical analysis was carried out using GraphPad Prism 7.0 software (https://www.graphpad.com/) and significance was calculated using non-parametric Student’s *t*-test. Data of two groups were compared using a two-sample *t*-test and *p*-value < 0.05 was considered statistically significant. Data are represented as mean ± SD from three independent experiments unless otherwise mentioned. Regression analysis was performed for the evaluation of IC_50_ values (curves with R^2^ ≤ 0.95 were accepted for analysis).

## 5. Conclusions

Taken together, our study revealed that GS treatment of CRC cells elicits novel proteomic signatures. Furthermore, GS significantly reduced the cell proliferation/migration, cell growth, and metabolism, carcinogenesis, as well as DNA replication whereas enhanced the process of exocytosis/tumor suppression. GS mediated inhibition of cell proliferation has been found to be correlated with induction of intrinsic apoptotic cell death in CRC cells. Our results provide a comprehensive overview of the mechanism of action of GS for its anticancer effects which could aid its beneficial use as a therapeutic agent in future for clinical applications.

## Figures and Tables

**Table 1:** Proteins dysregulated by guggulsterone treatment in HCT 116 cells identified by label-free shotgun proteomics approach.

Uniprot Accession	Gene Symbol	Protein Name	Fold Change	*p*-Value (−log_10_)
R4GNH2	*FBXO2*	F-box only protein 2	−1.5	1.6580
I3L1P8	*SLC25A11*	Mitochondrial 2-oxoglutarate/malate carrier protein	−1.5	1.3996
Q9UL25	*RAB21*	Ras-related protein Rab-21	−1.7	1.3732
P52292	*KPNA2*	Importin subunit alpha-1	−1.9	1.8005
M0R0G9	*SNRPA*	U1 small nuclear ribonucleoprotein A	−2.0	1.5435
O14744	*PRMT5*	Protein arginine N-methyltransferase 5	−2.0	1.9611
E9PES6	*HMGB3*	High mobility group protein B3	−2.1	1.4630
Q03135	*CAV1*	Caveolin-1	−2.1	1.3701
P27694	*RPA1*	Replication protein A 70 kDa DNA-binding subunit	−2.3	1.6471
P31153	*MAT2A*	S-adenosylmethionine synthase isoform type-2	−2.5	2.5716
P00491	*PNP*	Purine nucleoside phosphorylase	−2.6	2.3222
P25205	*MCM3*	DNA replication licensing factor MCM3	−4.6	1.8156
O75369	*FLNB*	Filamin-B	3.1	3.1921
P20073	*ANXA7*	Annexin A7	2.3	2.1067

Statistical significance of protein quantification was calculated using two-tailed Student’s *t*-test with permutation-based FDR of 5%.

## Supplementary Materials

The following are available online at https://www.mdpi.com/2072-6694/11/10/1478/s1, Figure S1: Proteomics data quality control. (A,B) Correlation plots using the LFQ intensity values obtained for each of the proteins identified by protein profiling and label-free quantification. Red, blue and green dots indicate the level of intensity as high, medium and low respectively. Pearson correlation analysis of replicate samples from untreated samples (A) and treated samples (B) with Pearson’s r value close to 0.9 reflects the quality of repeatability of replicate proteomics experiments. Figure S2: Workflow of mass spectrometry-based label-free shotgun proteomics approach. Step 1 involves the sample preparation wherein the protein lysates obtained from untreated and GS treated cells is separated by 1-D SDS-PAGE, stained by Coomassie blue stain solution followed by fractionation of proteins from the lanes by band cutting. Each of these bands are further destained, reduced by DTT, alkylated by IAA and in-gel digested by the protease enzyme trypsin. In step 2 the tryptic peptides are cleaned by passing through a C18 column by solid phase extraction, peptides are eluted and concentrated by vacuum centrifugation. The peptides are then diluted in solvent (5% formic acid) and injected into Liquid Chromatography system coupled to mass spectrometry. The peptides are separated by LC prior to their identification by mass spectrometry. In step 3, the MS data are processed using MaxQuant software for protein identification and quantification using label-free approach. The data from MaxQuant are further processed using Perseus for statistical significance and data interpretation. The significantly dysregulated proteins are further subjected to functional enrichment analysis using FunRich tool for their functional annotations and categorization. Finally, in Step 4 the data obtained from proteomics analysis are interpreted by visual representations/plots. Figure S3: Human apoptosis signaling array C1 (Raybiotech) coordinates with the corresponding target protein names (as extracted from the manufacturer’s data sheet). Each antibody is spotted in duplicates in the membrane. POS: Positive control spot, NEG: negative control spot as defined by the manufacturer. Figure S4: Human Proteome profiler apoptosis array (R&D) coordinates. Each antibody is spotted in duplicates in the membrane and the corresponding target protein names are as below. Table S1: Excel data file with the list of dysregulated proteins in GS treated HCT 116 cells obtained by proteomics analysis. Table S2: Excel data file generated after functional enrichment analysis of dysregulated proteins by FunRich tool with all the categorical classifications: Cellular component, Biological Process, Molecular function, and Biological pathways.
