# Supplementary material for: Protein Expression Profiling Identifies Key Proteins and Pathways Involved in Growth Inhibitory Effects Exerted by Guggulsterone in Human Colorectal Cancer Cells
Source: Cancers (Basel). 2019 Oct 1;11(10):1478. doi: 10.3390/cancers11101478 (PMC6826505; doi:10.3390/cancers11101478)
Supplement: Supplementary file 1 [file cancers-11-01478-s001.zip › 10.1 - cancers-591315 - Supplementary materials/cancers-591315 - Supplementary materials .docx]

Supplementary Materials: Protein Expression Profiling Identifies Key Proteins and Pathways Involved in Growth Inhibitory effects Exerted by Guggulsterone in Human Colorectal Cancer Cells


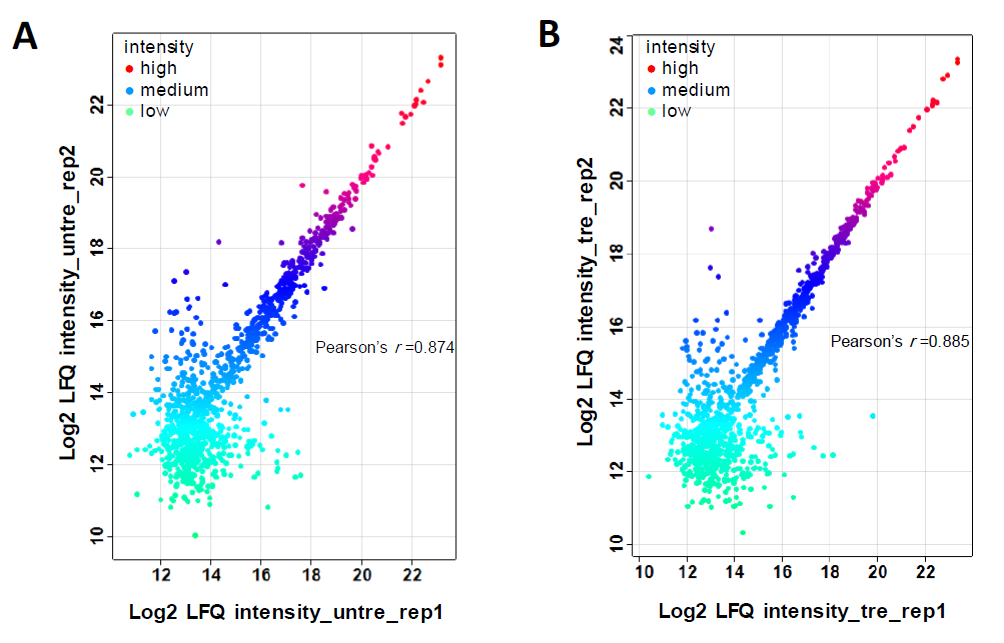


**Figure S1.** Proteomics data quality control. (A, B) Correlation plots using the LFQ intensity values obtained for each of the proteins identified by protein profiling and label-free quantification. Red, blue and green dots indicate the level of intensity as high, medium and low respectively. Pearson correlation analysis of replicate samples from untreated samples (A) and treated samples (B) with Pearson’s r value close to 0.9 reflects the quality of repeatability of replicate proteomics experiments.


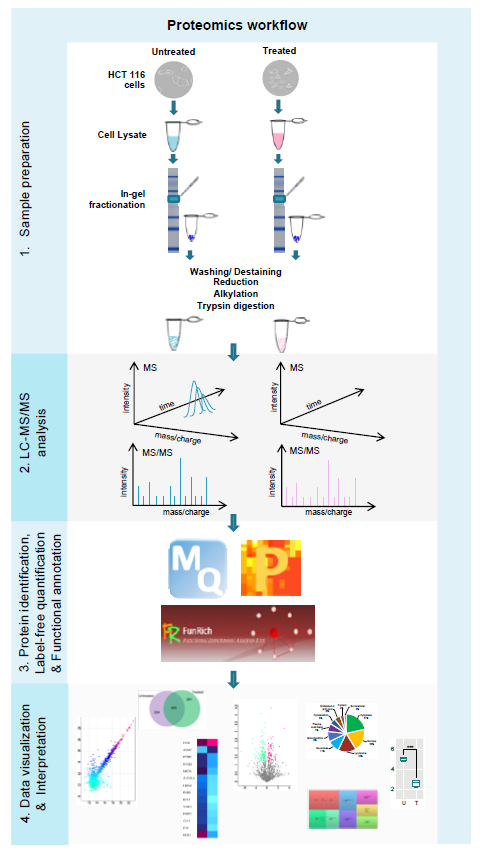


**Figure S2.** Workflow of mass spectrometry based label-free shotgun proteomics approach. Step 1 involves the sample preparation wherein the protein lysates obtained from untreated and GS treated cells is separated by 1-D SDS-PAGE, stained by Coomassie blue stain solution followed by fractionation of proteins from the lanes by band cutting. Each of these bands are further destained reduced by DTT, alkylated by IAA and in-gel digested by the protease enzyme trypsin. In step 2 the tryptic peptides are cleaned by passing through a C18 column by solid phase extraction, peptides are eluted and concentrated by vacuum centrifugation. The peptides are then diluted in solvent (5% formic acid) and injected into Liquid Chromatography system coupled to mass spectrometry. The peptides are separated by LC prior to their identification by mass spectrometry. In step 3, the MS data are processed using MaxQuant software for protein identification and quantification using label-free approach. The data from MaxQuant are further processed using Perseus for statistical significance and data interpretation. The significantly dysregulated proteins are further subjected to functional enrichment analysis using FunRich tool for their functional annotations and categorization. Finally in Step 4 the data obtained from proteomics analysis are interpreted by visual representations/ plots.


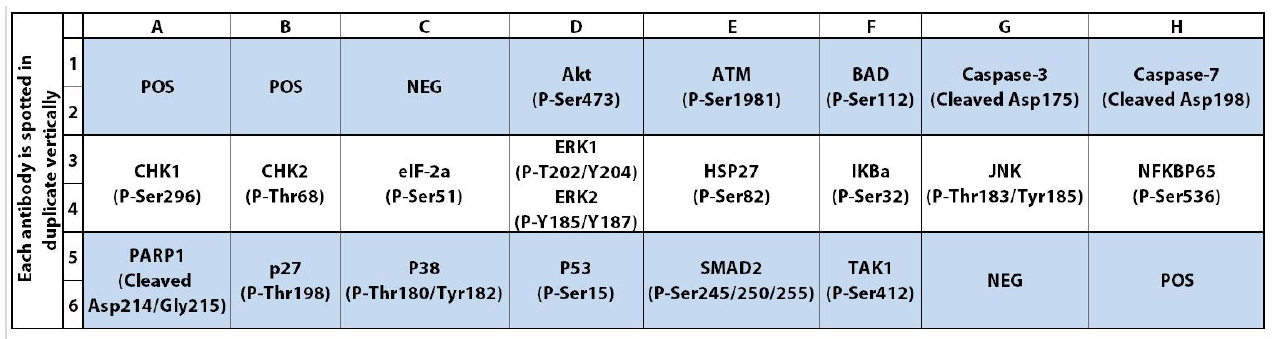


**Figure S3.** Human Proteome profiler apoptosis array (R&D) coordinates. Each antibody is spotted in duplicates in the membrane and the corresponding target protein names are as below.


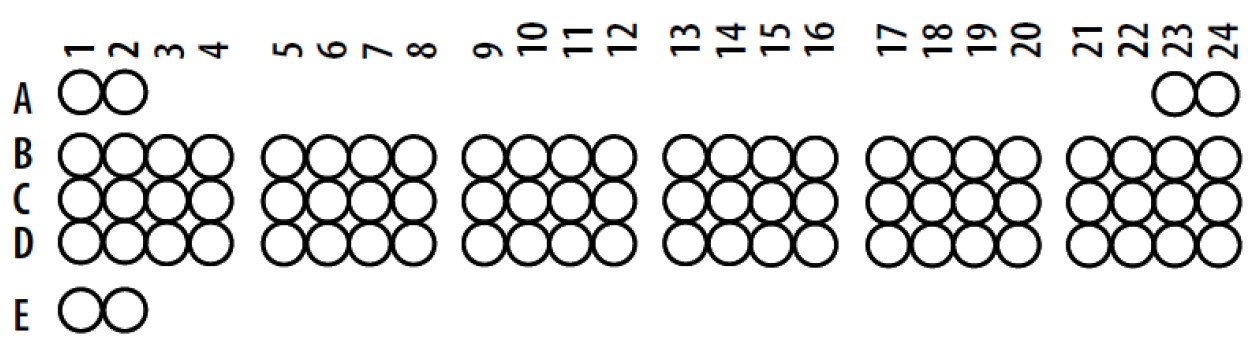


**Figure S4.** Human Proteome profiler apoptosis array (R&D) coordinates. Each antibody is spotted in duplicates in the membrane and the corresponding target protein names are as below.

A1, A2 Reference Spots A23, A24 Reference Spots

B1, B2 Bad B3, B4 Bax

B5, B6 Bcl-2 B7, B8 Bcl-x

B9, B10 Pro-Caspase-3 B11, B12 Cleaved Caspase-3

B13, B14 Catalase B15, B16 cIAP-1

B17, B18 cIAP-2 B19, B20 Claspin

B21, B22 Clusterin B23, B24 Cytochrome c

C1, C2 TRAIL R1/DR4 C3, C4 TRAIL R2/DR5

C5, C6 FADD C7, C8 Fas/TNFRSF6/CD95

C9, C10 HIF-1α C11, C12 HO-1/HMOX1/HSP32

C13, C14 HO-2/HMOX2 C15, C16 HSP27

C17, C18 HSP60 C19, C20 HSP70

C21, C22 HTRA2/Omi C23, C24 Livin

D1, D2 PON2 D3, D4 p21/CIP1/CDKN1A

D5, D6 p27/Kip1 D7, D8 Phospho-p53 (S15)

D9, D10 Phospho-p53 (S46) D11, D12 Phospho-p53 (S392)

D13, D14 Phospho-Rad17 (S635) D15, D16 SMAC/Diablo

D17, D18 Survivin D19, D20 TNF RI/TNFRSF1A

D21, D22 XIAP D23, D24 PBS (Negative Control)

E1, E2 Reference Spots

| 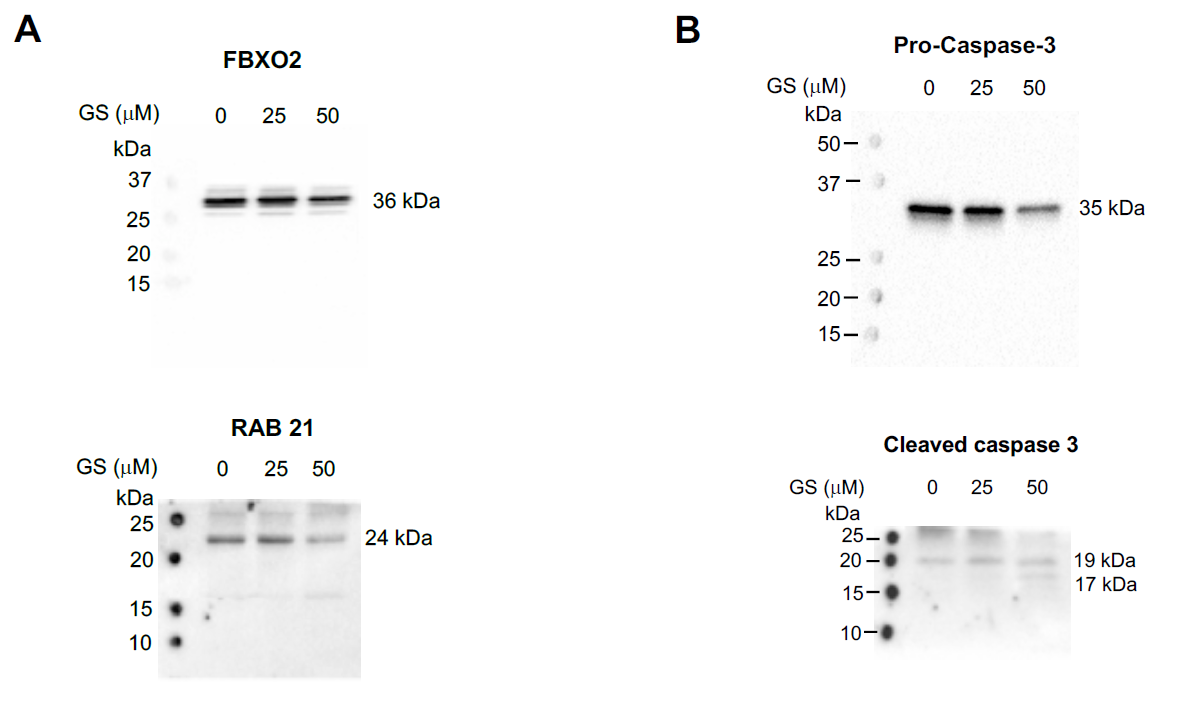 |
| --- |
| 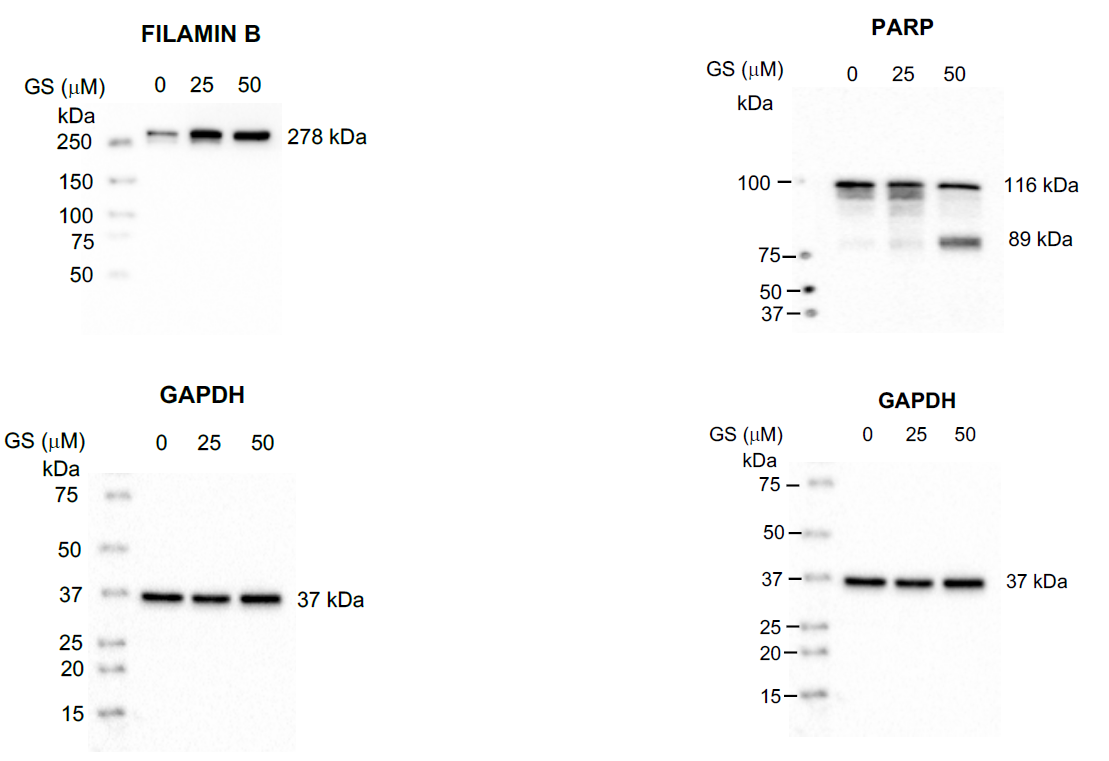 |

**Figure S5.** Validation by western blot analysis. (**A,B**) HCT 116 cells were treated with increasing doses of GS (asindicated) for 48 h and cells were lysed for western blot analysis with the antibodies **(A):** FBXO2, RAB21, Filamin B;**(B):** pro-caspase-3, cleaved caspase 3, and PARP. GAPDH served as the loading control.
